# Evidence of IgE-Mediated Cross-Reactions between *Anisakis simplex* and *Contracaecum osculatum* Proteins

**DOI:** 10.3390/pathogens10080950

**Published:** 2021-07-28

**Authors:** Foojan Mehrdana, María Lavilla, Per Walter Kania, Miguel Ángel Pardo, María Teresa Audicana, Natividad Longo, Kurt Buchmann

**Affiliations:** 1 Laboratory of Aquatic Pathobiology, Veterinary and Animal Sciences, Faculty of Health and Medical Sciences, University of Copenhagen, 1870 Frederiksberg C, Denmark; foojan@sund.ku.dk (F.M.); pwk@sund.ku.dk (P.W.K.); 2 AZTI-BRTA, Food Research, Parque Tecnológico de Bizkaia, Astondo Bidea-Edificio 609, E-48160 Derio, Spain; mlavilla@azti.es (M.L.); mpardo@azti.es (M.Á.P.); 3 Allergy Department, Araba Integrated Health Organization, Bioaraba.Osakidetza, Basque Health Service, Jose Atxotegui s/n, E-01009 Vitoria, Spain; MARIATERESA.AUDICANABERASATEGUI@osakidetza.eus (M.T.A.); MARIANATIVIDAD.LONGOARESO@osakidetza.eus (N.L.)

**Keywords:** *Anisakis simplex*, *Contracaecum osculatum*, allergens, human IgE, freshwater parasites, cross-reactions

## Abstract

Fish consumers may develop allergic reactions following the ingestion of fish products containing nematode larvae within the genus *Anisakis*. Sensitized patients may cross-react with proteins from insects, mites and mollusks, leading to allergic reactions even in the absence of the offending food. Potential cross-reactivity in *Anisakis*-allergic patients with larval proteins from other zoonotic parasites present in freshwater and sea fish should be investigated due to an increasing occurrence in certain fish stocks, particularly *Contracaecum osculatum*. In this work, we evaluated IgE-cross reactions by in vivo (skin prick tests with parasites extracts) and in vitro methods (IgE-ELISA and IgE-immunoblot). In vivo skin prick tests (SPT) proved the reactivity of *Anisakis*-sensitized patients when exposed to *C. osculatum* antigens. Sera from *Anisakis*-sensitized patients confirmed the reaction with somatic antigens (SA) and excretory/secretory proteins (ES) from *C. osculatum*. Only anecdotal responses were obtained from other freshwater worm parasites. Consequently, it is suggested that *Anisakis*-sensitized humans, especially patients with high levels of specific anti-*Anisakis* antibodies, may react to *C. osculatum* proteins, possibly due to IgE-mediated cross-reactivity.

## 1. Introduction

Third-stage larvae (L3) of *Anisakis simplex*, a member of the nematode family Anisakidae, use a variety of marine fish species as transport hosts and marine mammals as final hosts. The ingestion of fish products carrying live nematode larvae is considered a public health issue as it may cause gastrointestinal disease (anisakiasis), and also triggers allergic reactions [1]. Allergic forms of the disease are associated with IgE-mediated hypersensitivity, including urticaria, angioedema and anaphylaxis [2].

*Anisakis simplex* antigens are known to cross-react with other allergens from other ascarid nematodes [3,4]. In addition, the somatic allergens Ani s 2 and Ani s 3 have been reported to cross-react with molecules from mites, crustaceans and mollusks [5,6,7,8]. Consequently, although this fact has not yet been described, it cannot be excluded that some cross-reactivity may also occur between proteins from other closely related anisakid nematodes, such as those belonging to the genera *Pseudoterranova* or *Contracaecum*. In particular, third-stage larvae of a species within the latter genus, *C. osculatum* (liver worm), have recently been reported to parasitize the liver of Atlantic cod (*Gadus morhua*) from the Baltic Sea with a prevalence of up to 100% [9,10], while the prevalence of *Anisakis* in this fish is much lower [11,12]. It is therefore relevant to analyze whether proteins from *C. osculatum* cross-react with *Anisakis* allergens, since the potential allergenicity of this parasite has not been described previously. The objective of this work was to assess the potential cross-reactivity of *C. osculatum* proteins with *A. simplex* sensitized patients. Several additional parasites of freshwater fish (trematodes) were also studied, since their proteins could act as a hidden source of allergens in fish.

## 2. Materials and Methods

### 2.1. Parasites and Protein Preparations

*C. osculatum* third-stage larvae were isolated from Atlantic cod livers (a Baltic subpopulation of *Gadus morhua*) caught by a local fisherman in the Southern Baltic Sea. The livers were kept at 3–5 °C and immediate isolation of worm larvae was performed by artificial digestion (pepsin/HCl/NaCl solution with magnetic stirring at 250 rpm at 37 °C, using a volume of 10 mL solution per gram of fish liver). Following full digestion of the livers (1–2 h), the digest was filtered through a 300 µm sieve, whereafter nematodes were collected.

*A. simplex* larvae (non-encapsulated) were collected from the body cavity of freshly caught North Sea herring (*Clupea harengus*) provided by a local fish shop (Frederiksberg, Denmark).

Trematodes (*Tetracotyle* sp., *Apophallus* sp. and *Posthodiplostomum* sp.) were collected from several freshwater fish provided by different local fishermen.

All the parasites above were identified by standard morphological methods. Total extracts (TE) were obtained from all parasites using the technique adapted from Perteguer et al. [13].

Excretory/secretory proteins (ES) from *A. simplex* and *C. osculatum* were collected as described previously [14,15]. In brief, worm larvae were washed several times in phosphate buffered saline (PBS, Dacos A/S, Esbjerg, Denmark) and then incubated at 37 °C in sterile 12-well NunclonTM cell culture plates (WVR, Denmark) for five days. Each well contained 10 live larvae in 2.5 mL PBS with antibiotics (200 µg/mL ampicillin and 400 µg/mL kanamycin sulphate) (Sigma-Aldrich, St. Louis, MO, USA). After incubation, the medium was filtered through 0.20 µm Minisart^®^ filters (Sigma-Aldrich) and stored at −40 °C until further use.

In order to isolate somatic proteins (SA), recovered parasite larvae were homogenized for 4 min at a frequency of 20 Hz in a TissueLyser II (Qiagen, Hilden, Denmark) in PBS-containing protease inhibitors (5 mM ethylenediaminetetraacetic acid, 1 mM phenyl-methyl-sulphonyl fluoride and 0.1 trypsin inhibitor unit/mL aprotinin) [3]. The homogenates were centrifuged at 16,000 rpm for 30 min at 4 °C and the supernatant was collected and preserved at −20 °C until use.

### 2.2. Patient Sera

Sera from 9 patients admitted to the Araba University Hospital (Allergy Department, Vitoria, Spain) with diagnosed *A. simplex* allergy (but not reporting previous anisakidosis or *Anisakis* infestation, according to their clinical records), and presenting high levels of specific anti-*Anisakis* IgE (higher than 30 kUA/L) (ImmunoCAP FEIA System, ThermoFisher Scientific, Waltham, MA, USA) were collected. These sera were mixed (by equal volume of each serum), and this pool was used for specificity in vitro testing.

Negative control sera were obtained from healthy individuals without any allergy symptoms (2 patients) and from patients allergic to other food proteins but not to fish or *A. simplex* (10 patients). These patients were tested by skin prick tests (SPT) with an *A. simplex* extract with a negative result before using their sera for in vitro tests. Sera were tested later (in vitro tests) in two different pools (one for non-allergic and one for those allergic to other proteins).

### 2.3. Evaluation of Cross-Reactivity

This work has been approved by the Ethics Committee for Clinical Research (CEIC) of the Araba Hospital (Vitoria, Spain) with internal ref. PI2018118. All participant patients gave written informed consent and patient anonymity has been secured through the entire study.

#### 2.3.1. Skin Prick Tests

The skin prick tests (SPT) comprised two groups of *A. simplex*-sensitized individuals voluntarily recruited from local patients with an allergic reaction induced by the ingestion of fish dishes. Group 1: six patients classified as “low IgE” (L); sensitized but without allergic symptoms; positive SPT with *A. simplex* extract and positive low specific-IgE to *A. simplex* (<2.5 kUA/L). These patients had negative SPT and negative specific-IgE against fish extracts. Group 2: nine patients classified as “high IgE” (H), showing allergic symptoms, positive SPT with *A. simplex* extract and positive high specific-IgE to *A. simplex* (media value of 41.0 kUA/L of *A. simplex*-specific IgE, interquartile range: 30.5 to 62.9 kUA/L). These patients had negative SPT and negative specific-IgE against fish extracts. Two non-allergic patients were evaluated as negative controls.

SPTs were performed in duplicate on the volar surface of each forearm by using a standard 1 mm tip lancet (ALK-Abelló), following the EAACI recommendations [16]. Histamine hydrochloride (10 mg/mL) and physiological serum (PSS) were used as positive and negative controls, respectively. Tests were performed with complete protein extracts (TE) from all species. The wheal areas were measured by assessing the diameters in millimeters (mm) and wheal diameters >3 mm were considered positive reactions.

#### 2.3.2. ELISA

For the in vitro evaluation of cross-reactions between *A. simplex* and *C. osculatum* antigens, microtiter plates (microwell F96 PS Maxisorp, NUNC) were coated overnight at 4 °C with 100 µL of a solution containing 5 µg/mL of the corresponding antigens: *A. simplex* (SA or ES) or *C. osculatum* (SA or ES) diluted in coating buffer (15 mM Na_2_CO_3_, 35 mM NaHCO_3_, sodium carbonate/bicarbonate buffer, pH 9.6). At this point, additional negative controls were also included to check for non-specific IgE-binding to wells by including wells with only a blocking buffer (no antigen). After blocking with 1% (*w*/*v*) bovine serum albumin (BSA, A7906, Sigma-Aldrich, St. Louis, MO, USA) in PBS (150 mM NaCl, 8 mM K_2_HPO_4_, 16 mM KH_2_PO_4_, pH 7.4) for 1.5 h at room temperature (RT), the plates were incubated with pooled sera (each sample tested in quadruplicate) diluted 1:100 in PBS for 1 h at 37 °C. This dilution was selected based on prior optimization of the assay (data not shown). The plates were subsequently incubated with 100 µL of a goat anti-human IgE-HRP (A9667 Sigma-Aldrich, St. Louis, MO, USA) (diluted 1:4000 in PBS) for 40 min at 37 °C. Unbound proteins were washed three times using PBST (PBS with 0.05% (*w*/*v*) Tween-20) between all the steps. A color reaction was developed by adding 100 µL of tetramethylbenzidine (1-STEP^TM^ Ultra TMB-ELISA SUBSTRATE, Thermo Scientific™) and stopped with 50 µL of 2 M H_2_SO_4_. Optical density (OD) was measured at 450 nm using a microplate reader Thermo Scientific™ Varioskan Flash (Waltham, MA, USA).

#### 2.3.3. Gel Electrophoresis and Immunoblot

Proteins were electrophoretically separated under reducing conditions on NuPAGE^®^Novex^®^Bis-Tris Gels (4–12% polyacrylamide) using an XCell SurelockTM Mini-Cell (Invitrogen, Carlsbad, CA, USA) according to the manufacturer’s instructions. Samples contained 54 and 30 µg protein for SA and ES isolates, respectively. Separated proteins were transferred onto a nitrocellulose membrane in an XCell II Blot Module running at 30 V for 1 h. Subsequently, the membrane was blocked in Tris-buffered saline containing 0.05% (*v*/*v*) Tween 20 (TBS-T, pH 7.6) and 5% (*w*/*v*) skimmed milk powder (SMP), whereafter it was incubated in human sera (diluted 1:4 with TBS-T and 5% (*v*/*v*) SMP) overnight at RT. Following each step, the membrane was washed (3 × 10 min) with TBS-T. The next incubation applied a monoclonal mouse anti-human IgE (diluted 1:250, clone GE-1, ascites fluid, Sigma-Aldrich) for 2 h under agitation at RT. Following a washing step, the membrane was incubated in the goat anti-mouse IgG conjugated with HRP (diluted 1:500, Sigma-Aldrich) (2 h agitation at RT). After a final wash, reactivity was visualized by adding substrate (AEC staining kit, Sigma-Aldrich).

### 2.4. Statistical Analysis

ELISA results were analyzed with two-way ANOVA followed by Tukey’s Multiple Comparison test using GraphPad Prism version 7 and *p*-values < 0.05 were considered statistically significant.

## 3. Results

Skin prick tests (SPT) showed that an important cross-reactivity may occur between proteins from *Anisakis* and *Contracaecum* parasite species (Table 1), while only anecdotal responses (minor responses in three patients, in all cases less intense than the histamine control and the target species of this work) were obtained from other freshwater worm parasites (data not shown).

*Anisakis*-sensitized patients reacted strongly both to *A. simplex* and *C. osculatum* proteins. However, there are differences in the level of reactivity depending on the patient sensitivity: as can be seen in Table 1, those patients with high levels of specific IgE to *Anisakis* (H) reacted to *C. osculatum* to a greater extent (seven of nine patients) than patients sensitized to *Anisakis* with lower IgE levels (L), who also showed a lower reactivity to *C. osculatum* (two of six patients).

**Figure 1:**
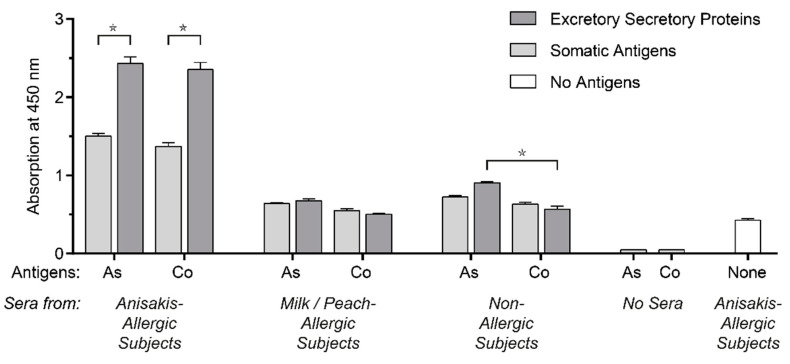
Binding of serum IgE to excretory/secretory proteins (dark gray columns) and somatic antigens (soft gray columns) from *A. simplex* (As) and *C. osculatum* (Co) measured by ELISA: (1) pooled serum from patients sensitized to *A. simplex*; (2) patients allergic to other food allergens; (3) non-allergic subjects. *: *p* < 0.0001. Other negative controls (white columns) are also included.

In vitro tests confirmed these results: ELISA results showed that human IgE in sera from *Anisakis* sensitized patients also binds to *Contracaecum* somatic antigens (SA) and excretory/secretory proteins (ES) components (Figure 1). No statistically significant difference in binding strength of IgE was recorded with regard to SA (*p* = 0.61) and ES proteins (*p* = 0.88) from the two worm species. Additionally, it was noted that the IgE-binding was significantly higher to ES proteins than to SA proteins in both *A. simplex* and *C. osculatum* species (1.6 times, *p* < 0.0001 and 1.7 times, *p* < 0.0001, respectively) (Figure 1). Residual IgE-binding was shown to parasite antigens when sera from other patients (non-allergic or allergic to other foods) were used, probably due to unspecific binding; nonetheless, it does not interfere with aforementioned specific reactions to parasites proteins.

**Figure 2:**
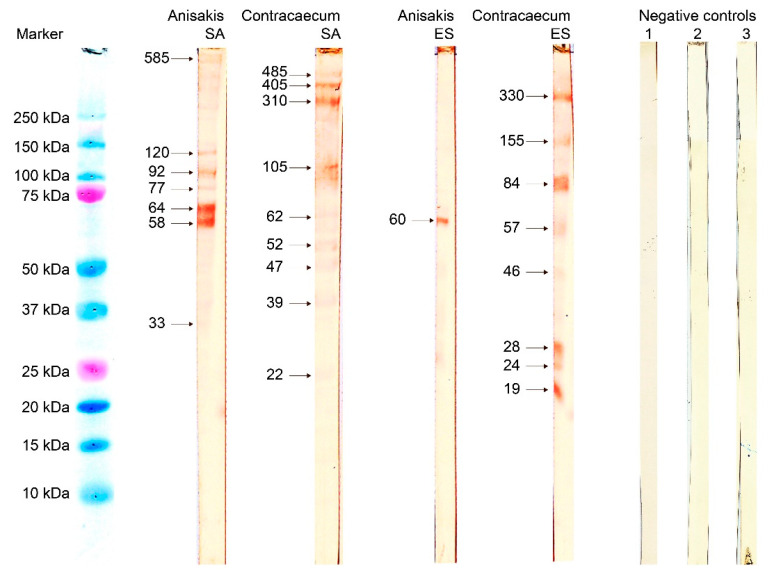
Immunoblot with isolated SA and ES proteins from *A. simplex* and *C. osculatum* third-stage larvae separated by SDS-PAGE (reducing conditions) and transferred to nitrocellulose. Reactions with IgE from pooled serum samples from patients sensitized to *A. simplex*. Marker: Precision Plus Protein^TM^ Dual Color Standards.

Finally, Western blotting (WB) verified that IgE in sera from *A. simplex* sensitized patients bound to specific SA and ES antigens from both *A. simplex* and *C. osculatum* (Figure 2): The sera pool detected several SA from both parasites. Contrarily, only one dominant protein band (approximately MW 60 kDa) was evidenced from the *Anisakis* ES sample, whereas additional IgE-binding components were detected among *Contracaecum* ES proteins (MW from 19 to 330 kDa). Sera from non-allergic patients did not recognize any specific parasite protein (Figure 2).

## 4. Discussion

It is well documented that *A. simplex* produces a number of allergenic proteins which are involved in its immunopathology [2,14,17]. As said previously, *Anisakis simplex* antigens are known to potentially cross-react with other allergens from, e.g., other ascarid nematodes [3,4] and with molecules from mites, crustaceans and mollusks [5,6,7,8]. However, although information on potential similar allergens in other anisakid nematodes has been hypothesized [18,19], and IgG antigenic homology among nematodes has been described previously [3,18], in vivo and IgE cross-reactions between fish parasites within this family had not yet been studied. To our knowledge, this is the first time that IgE cross-reactivity between two related species, *Anisakis simplex* and *Contracaecum osculatum*, both present in fish, has been confirmed in some patients.

It is not known if fish products carrying *C. osculatum* larvae (live or killed) are able to induce IgE production in consumers. Relatively few infections of humans with this parasite have been published [20,21,22,23] and mice studies have indicated reduced allergenicity of *Contracaecum* proteins [24]. Moreover, the present study was performed with patients from Spain, where infections with *Contracaecum* have never been recorded [14,25]. All these facts indicate that the observed cross-reactivity by both in vivo and in vitro methods is due to a real potential cross-reactivity between the two parasites and not because of the presence of specific IgE against *Contracaecum* proteins in allergic patients’ sera.

In our previous studies of allergy to *Anisakis simplex*, a correlation was observed between high levels of specific IgE, a high repertoire of bands recognized in Western blot and the severity of allergic reactions and anaphylaxis (severe allergy) [2]. This fact could explain why patients with high levels of specific IgE to *Anisakis* reacted more frequently and to a greater extent to *C. osculatum*, and is also in agreement with in vitro ELISA and WB results (performed with sera with high *A. simplex*-specific IgE), where important cross-reactivity of IgE with *Contracaecum* proteins were evidenced.

Regarding ELISA results, the higher level of reactivity against ES proteins, when analyzed at the same concentration and conditions than SA proteins, may be due to a higher affinity to specific IgE to *A. simplex* ES compared to SA allergens [26]. Additionally, the similar reaction to ES proteins observed for both parasites would suggest a high similarity between *Anisakis* and *Contracaecum* IgE-epitopes, since specific *anisakis*-IgE generally reacts more specifically to these kind of proteins [5,27,28,29]. This fact would be also confirmed by the numerous *Contracaecum* protein bands that were revealed in the WB analysis, recognized by specific *Anisakis* IgE from sera in this work, and is in agreement with previous works by IgG-ELISA-based serodiagnosis [18] and by proteomic profiling [19] with both species. However, personal responses have not been studied in this work by in vitro methods, and it should be stated that the individual response against *A. simplex* and *C. osculatum* antigens could differ depending on the individual patients with allergic anisakidosis, as observed by in vivo methods.

There is enough evidence that purified allergens from *A. simplex* are potent enough to cause anaphylaxis in some individuals even as a result of a skin prick test with an extract of the parasite, and that allergens are also present in the flesh of fish in areas close to the larvae. In the last 20 years, it has become apparent that *A. simplex* is one of the most important hidden food allergens in the adult population suffering acute urticaria and anaphylaxis [30]. Regarding the results presented in this and previous works [19], and the practical lack of SPT-response from the other studied parasites from freshwater (*Tetracotyle* sp., *Apophallus* sp. and *Posthodiplostomum* sp.), attention should be focused subsequently on *Contracaecum* sp. and very closely related parasites, especially in order to understand future clinical situations or diagnosis/detection test developments.

We are still lacking adequate knowledge about proteins released by parasites within the genus *Contracaecum* and their allergenic potential, if any, which should be investigated in future studies. Other parasites may elicit IgE hypersensitivity [17,31], but in the case of *Contracaecum* sp., no triggering of specific IgE or IgG production against SA or ES antigens have been evidenced in BALB/c mice with live larvae [24]. Nevertheless, only a few studies have investigated the occurrence of IgE-induced allergy to fish parasites except for *A. simplex*.

## 5. Conclusions

Our findings call for further studies on *C. osculatum* allergens for elucidation of any potential allergenicity of this parasite. Additionally, due to the suggestion that *C. osculatum* proteins may elicit reactions in some sensitized consumers, the implications of these cross-reactions in relation to individualized responses, clinical significance and the development of specific diagnosis/detection tests with an immunological base should be thoroughly analyzed. Regarding food safety, the stability of antigens of this parasite when exposed to various heat and freeze processing methods commonly applied by the industry and consumers should also be examined. This information is still needed in order to understand future clinical situations as well as to establish risk assessments and, if needed, food control and safety recommendations.

## Figures and Tables

**Table 1:** Wheal size (length × width in mm) obtained by skin prick test (SPT) in patients allergic to *Anisakis* with low levels (L) of specific anti-*Anisakis* IgE (<2.5 kUA/L) or high levels (H) of specific anti-*Anisakis* IgE (>30 kUA/L). N: negative (wheal size length and width less than 3 mm).

		Right Arm		Left Arm	
Patient ID	IgE Level	*Contracaecum*	*Anisakis*	*Contracaecum*	*Anisakis*
L1	1.2	N	4 × 3	N	5 × 4
L2	2.3	N	4 × 3	N	4 × 4
L3	1.1	N	N	N	N
L4	0.9	3 × 4	3 × 3	4 × 4	4 × 4
L5	1.2	6 × 4	5 × 3	N	7 × 4
L6	1.1	N	3 × 3	N	5 × 6
H1	62.9	6 × 4	4 × 4	5 × 4	6 × 4
H2	54.3	6 × 5	6 × 5	7 × 5	5 × 5
H3	40.7	N	12 × 6	N	10 × 6
H4	39.6	3 × 3	10 × 6	4 × 5	12 × 7
H5	38.1	7 × 4	7 × 5	7 × 5	7 × 5
H6	37.4	4 × 4	6 × 5	5 × 3	9 × 7
H7	34.6	3 × 4	13 × 6	3 × 3	14 × 6
H8	31.3	9 × 7	10 × 7	8 × 7	10 × 7
H9	30.5	N	3 × 3	N	3 × 5

## Data Availability

Data supporting the analyses are available on reasonable request from the corresponding authors.
